# Estradiol and Progesterone Regulate the Migration of Mast Cells from the Periphery to the Uterus and Induce Their Maturation and Degranulation

**DOI:** 10.1371/journal.pone.0014409

**Published:** 2010-12-22

**Authors:** Federico Jensen, Mariana Woudwyk, Ana Teles, Katja Woidacki, Florin Taran, Serban Costa, Sara Fill Malfertheiner, Ana Claudia Zenclussen

**Affiliations:** 1 Experimental Obstetrics & Gynecology, Medical Faculty, Otto-von-Guericke University, Magdeburg, Germany; 2 Laboratory of Histology and Embryology, School of Veterinary Sciences, National University of La Plata, La Plata, Buenos Aires, Argentina; 3 Doctoral Program in Experimental Biology and Biomedicine, Center for Neuroscience and Cell Biology, University of Coimbra, Coimbra, Portugal; 4 University Women's Clinic, Otto-von-Guericke University, Magdeburg, Germany; Institut Jacques Monod, France

## Abstract

**Background:**

Mast cells (MCs) have long been suspected as important players for implantation based on the fact that their degranulation causes the release of pivotal factors, e.g., histamine, MMPs, tryptase and VEGF, which are known to be involved in the attachment and posterior invasion of the embryo into the uterus. Moreover, MC degranulation correlates with angiogenesis during pregnancy. The number of MCs in the uterus has been shown to fluctuate during menstrual cycle in human and estrus cycle in rat and mouse indicating a hormonal influence on their recruitment from the periphery to the uterus. However, the mechanisms behind MC migration to the uterus are still unknown.

**Methodology/Principal Findings:**

We first utilized migration assays to show that MCs are able to migrate to the uterus and to the fetal-maternal interface upon up-regulation of the expression of chemokine receptors by hormonal changes. By using a model of ovariectomized animals, we provide clear evidences that also *in vivo*, estradiol and progesterone attract MC to the uterus and further provoke their maturation and degranulation.

**Conclusion/Significance:**

We propose that estradiol and progesterone modulate the migration of MCs from the periphery to the uterus and their degranulation, which may prepare the uterus for implantation.

## Introduction

In mammals, the successful implantation of the blastocyst into the endometrium and the subsequent development of a decidual cell response involve many interactions between the mother and the conceptus [1]. Uterine-derived histamine has long been suspected as a key regulator in implantation due to its ability of altering uterine vascular permeability and inducing stromal decidualization [2]. Histamine is produced mainly by MCs that are present in both the uterus and placenta [3]. Moreover, human preimplantated embryos induce MCs to release histamine by secreting histamine releasing factor [4].

The invasion of trophoblast cells into the maternal endometrial tissue is not the result of passive growth pressure but of an active biochemical process [5]–[7]. It necessarily involves the destruction and/or displacement of the basement membrane, extracellular matrix (ECM) and possibly cellular components of the maternal decidua. This process is regulated by a fine balance between the production of proteolytic pro-enzymes (in particular matrix metalloproteinases, MMPs), their physiological activators (e.g. plasmin) and their inhibitors (tissue inhibitors of metalloproteinases, TIMPs). The proteolytic enzymes, capable of digesting the different constituents (several collagen types, laminin, fibronectin) of the endometrial basement membrane/ECM, are considered the rate-limiting steps of the trophoblastic invasion [8]–[10]. The potential role of the MCs on mediating ECM degradation trough the activation and production of metalloproteinases MMPs has been highlighted [11]. Moreover, MC tryptases and chymases have been shown to activate the precursors of MMP2 [12], MMP9 [13], collagenase and stromelysin [14].

As MC precursors circulate through the vasculature and migrate into vascularized tissue, where they maturate and act before they will either regenerate or die, MC progenitors may migrate to the uterus in every menstrual/estrous cycle and expand upon pregnancy-specific stimuli or reach the uterus shortly after conception. After confirming the presence of MCs at the fetal-maternal interface and observing that they interact with uterine cells and trophoblasts, we concentrated on the mechanisms behind MC migration from the periphery to the uterus, which remained so far unexplored. Since it is known that in both, human menstrual and mouse estrus cycle respectively, the release of chemokines from the uterus occurs under hormonal influence [15]–[17] and that chemokines can attract cells, e.g. MCs, we postulated that female hormones, estradiol (E_2_) and progesterone (P_4_) modulate the expression of chemokine receptors on MC precursors from the periphery as a possible mechanism of MC migration to the uterus. We further tested the hypothesis whether female hormones modulate MC maturation and degranulation, which would prepare the uterus for a possible implantation because of the release of pivotal factors as histamine.

## Materials and Methods

### Animals

6 weeks old female ovariectomized (OVX) mice (C57B6/J) were purchased from Charles River (Germany). Mice were kept in our animal facility under optimal conditions in a 12 h light 12 h dark cycle. Chow and water were applied *ad libitum*. Animal experiments were carried out according to institutional guidelines after Ministerial approval (Reviewing board institution: Landesverwaltungsamt Sachsen-Anhalt upon evaluation and writing the approval (ID AZ2-868 to ACZ). The experiments were conducted in conformity with the European Communities Council Directive 86/609/EEC. Animals were treated subcutaneously with 5 µg E_2_ in 0.2 ml of NaCl 0.95% (n = 5) or 2.5 mg P_4_ in 0.2 ml NaCl 0.95% (n = 5) during 3 consecutive days. These groups were named E_2_ and P_4_ treated group respectively. An additional group of animals (n = 5) received 5 µg E_2_ in 0.2 ml of NaCl 0.95% during 2 consecutive days and on the third day they were treated with 2.5 mg P_4_ in 0.2 ml of NaCl 0.95%. This group is named E_2_+P_4_ treated group. Control animals (n = 5) received 0.2 ml of NaCl. Hormonal treatments were performed at 8:00 A.M. and the animals were sacrificed at 6:00 P.M. on the third day of treatment.

### Tissue collection and processing

The animals were sacrificed by cervical dislocation and the uterine horns were removed. One uterine horn was fixed in cold 96% ethanol for 24 h while the other one was snap-frozen in liquid nitrogen and further stored at −80°C for RNA extraction. The 96% ethanol-fixed tissues were dehydrated, embedded in paraffin following our standard protocol [18], cut into serial sections at 5 µm, and mounted onto coated slides. Sections were dewaxed in xylene (Sigma Chemical Co.), rehydrated and stained with 0.1% of toluidine blue for 1 min.

### Cells and cell culture

The human immature mast cell line, HMC-1, has been kindly provided by Dr. J.H. Butterfield (Mayo Clinic, Rochester, MN, USA) upon MTA agreement [19]. Cells were cultured in Iscove's modified Dulbecco's medium (IMDM; Cellgro, Kansas City, MO) supplemented with 10% iron-enriched calf serum (Hyclone, South Logan, UT) and 100 nM penicillin/streptomycin (Invitrogen). To avoid false positive results due to hormone presence in the serum used to enrich the culture medium, we used charcoaled fetal calf serum (FCS) and phenol red–free media (treatment medium), as previously described [20].

We developed primary cultures of bone marrow-derived mast cells (BMMCs) using femurs of virgin C57BL/6J female mice, as described elsewhere [21]. BMMC cultures contained >97% pure mast cells (MCs) after 5 weeks, as assessed by flow cytometry analysis of CD117 and FcεRIα.

For migration studies, first trimester trophoblast cells were isolated from placenta samples of women undergoing normal pregnancies which were legally interrupted (n = 3). This has been previously approved by the Ethics Committee of the Medical Faculty of the Otto-von-Guericke University, Magdeburg (EK28/08 to ACZ). All patients involved in this work were properly informed about the purpose of our research and gave their written consent before the sampling. Primary first trimester trophoblast cells were obtained as described elsewhere [22] and further cultured in Medium 199 (Invitrogen) supplemented with 10% FBS (Biochrom) and 50 mg/ml Normocin (Amaxa). The choriocarcinoma trophoblast cell line JEG-3 (Passage 27–30) and the human uterine cell line (AN3-CA) were purchased from Cell Line Service CLS, Germany. The human keratinocyte cell line HaCaT was kindly provided by Dr. Martina Seifert, Charité, Berlin. JEG-3 and HaCaT cells were cultured in DMEM normal growth medium (Invitrogen) supplemented with 10% FBS and 100 nM penicillin/streptomycin (Invitrogen). Cell cultures were maintained at 37°C and 5% CO_2_. AN3-CA cells were culture in MEM medium supplemented with 1% Non-Essential Amino Acids (NEAA), 1 mM sodium pyruvate, 1% streptomycin-penicillin and 10% FBS.

### Migration assays

Migration assays were performed as described elsewhere [23]. We analyzed the migration of HMC-1 cells towards either human first trimester trophoblasts, human choriocarcinoma cell line (JEG-3) or human uterine cell line (AN3-CA). Trophoblasts or uterine cells were trypsinized and plated overnight (ON) in 24-well plates (1×10^5^ cells/well) in their respective growth media. Medium of either JEG-3 or human primary trophoblast cells was changed to Opti-MEM (1 ml/well) and cell inserts (8 µm; BD Falcon) were placed into the wells. For uterine cells, medium was changed to Opti-MEM (1 ml/well) containing 100 pg/ml of E_2_ and 10 ng/ml of P_4_. Thereafter, HMC-1 cells (4×10^4^/well diluted in Opti-MEM) were filled in each insert. After 0, 4, 8, 24, or 48 h, the inserts were removed and supernatants from the upper and lower chambers were taken. Cells were washed with PBS and stained with anti-human FITC-CD117 (BD Pharmingen) for determination of total number of MCs. After ON incubation with 1% paraformaldehyde solution (PFA) (Carl Roth), the absolute number of HMC-1 present in the lower and upper compartments was determined by flow cytometry (FACSCalibur; BD Biosciences). The number of the spontaneously migrated cells in all cases was subtracted and the percentage of migrated cells was calculated using the following formula:
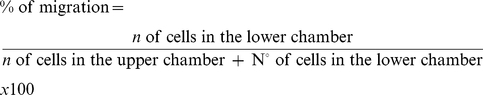



*n*For the migration of BMMCs towards CCL5 we used the same system detailed before and followed a published protocol [24]. Briefly, BMMCs (4×10^5^) were placed in the upper chamber of the system, and CCL5 (100 ng/ml) was added to chemotaxis buffer in the lower chamber. Cells were allowed to migrate for 3 h, recovered from the lower chamber, and counted by flow cytometry.

### Protein isolation, SDS-PAGE and Western blot

Protein extraction from cell lysates was performed on ice. Cells were re-suspended in lysis buffer (20 mM Tris-HCl, pH 8.0, 137 mM NaCl, 1% Nonidet P-40, and 10% glycerol) supplemented with protease inhibitors (0.5 mM PMSF, 0.025 mM N-CBZ-L-phenylalanine chloromethyl ketone, 0.025 mM N'-p-tosyl-lysine chloromethyl ketone, and 0.025 mM L-1-tosylamide-2-phenyl-ethylchloromethyl ketone) for 20 min. After incubation, samples were centrifuged at 12,000× g for 30 min at 4°C and the pellet was discarded. Protein content was determined with the Bradford assay (Bio-Rad) as indicated by the manufactures. Aliquots of proteins (10–20 µg) were resolved by SDS-PAGE 10% (Tryptase, estradiol receptor alpha (ERα) and estradiol receptor beta (ERβ)) or 8% (Progesterone Receptor (PR)) at 120 V for 1.5 h, and transferred into nitrocellulose membranes in transfer buffer containing 20% methanol (vol/vol), 0.19 M glycine, and 0.025 M Tris-base (pH 8.3). For the blot detection, mouse polyclonal anti-human tryptase (Millipore) (1∶1000), rabbit anti-human and mouse ERα, ERβ and PR (all diluted 1∶100, all from Santa Cruz, Biotechnology) were used as primary antibodies. The membranes were then incubated with an anti-mouse or anti rabbit biotin-conjugated IgG antibody diluted 1∶1000 (Dako) for 1 h at RT and then with avidin-horseradish peroxidase complex (ABC, Amersham) for 30 min. The chemiluminescence signal was generated with luminol (A8511-5G, Sigma-Aldrich), 4-hydroxycinnamic acid (p-coumaric acid; C9008-25G, Sigma-Aldrich), and hydrogen peroxide (Merck). Rainbow protein, molecular weight (m.w.) was from Amersham. The blots were exposed to medical x-ray film (CP-BU New, Agfa). Western blots were quantified using the ImageJ program (National Institute of Health, http://rsbweb.nih.gov/ij/). GAPDH served as house keeping gene.

### Mast cell degranulation assay and mast cell degranulation in vivo

For quantification of mast cell degranulation we used the “mast cell degranulation kit” (Millipore) in which tryptase activity is detected in the supernatant, following the manufacturer instructions. As controls, HMC-1 cells were stimulated for 1 h either with calcium ionophore (positive control) or calcium ionophore plus protamine inhibitor (negative control). For the quantification of MC degranulation *in vivo*, MCs with more than three granules outside of the cell shape or with empty cavities in the cytoplasm were considered to be degranulated [25].

### Flow cytometry

Human HMC-1 or mouse BMMCs (5×10^5^ cells/well) were plated in triplicate 24 h before treatment on 24-well plates with 1 ml of treatment medium (RPMI phenol red-free, 3% of charcoaled fetal calf serum (FCS), 1% penicillin/streptomicyn). HMC-1 were treated either with E_2_ (50, 100, 200 and 400 pg/ml), P_4_ (1, 5, 10 and 50 ng/ml) or E_2_+P_4_ (100 pµ/ml; 10 ng/ml) for 1 h. BMMC cells were treated either with E_2_ (10, 15, 20 and 30 pg/ml), P_4_ (1, 2, 4 and 8 ng/ml) or E_2_ + P_4_ (20 pµ/ml, 4 ng/ml) for 1 h. The concentrations used in this study are representative of the physiological concentrations of E_2_ and P_4_ through the menstrual or estrus cycle respectively. After treatment the cells were carefully washed with PBS and stained for chemokine receptors using the following anti-human antibodies: FITC-CCR5, PE-CCR4 or anti mouse PE-CCR5 and Alexa 647-CCR3 (BD, Bioscience). Negative controls for quadrant settings included FITC, PE or Alexa-labeled isotype antibodies as well as cells without any staining which was used for setting the gate. A total of 1×10^5^ events were measured in each case. Analysis by flow cytometry was performed using a FACS Calibur (BD, Bioscience).

### Immunofluorescence

Both, JEG-3 cell line and human primary trophoblast cells, were trypsinized and plated ON in a 4-well chamber slide Nunc-plate (Lab-Tek, Germany) at a concentration of 5×10^4^ cells/well in their respective growth media (Invitrogen). Medium was then changed to Opti-MEM medium (1 ml/well) containing 4×10^4^ HMC-1 cells and cultured for 24 h. The supernatant was harvested and the cells washed twice with PBS. After washing, the cells were fixed for 30 min in formalin (Roth, Germany). After blocking with 10% FBS in PBS the slides were incubated with polyclonal rabbit anti human CD117 cKit antibody, diluted 1∶100 (DAKO) for 1 h at RT. Secondary FITC anti rabbit IgG (Invitrogen) antibody diluted 1∶1000 was added for 1 h at RT. DAPI was chosen as counterstaining and the slides were mounted with Vectashield (VECTOR). Replacing the first antibody by PBS+BSA 1% or diluted rabbit serum 1∶100 served as negative control. For immunofluorescence staining of murine tissue we used cryosections which were analyzed for CD117^+^ cells using the same protocol employing a purified rat anti mouse CD117 monoclonal antibody diluted 1∶100 (Cederlane, Germany) and a FITC-goat anti rat antibody diluted 1∶1000 as secondary antibody (Invitrogen, Germany).

### Real Time RT-PCR

Frozen uterine tissues (100 mg) were treated with 1 ml of TRIzol (Invitrogen) and disaggregated with a homogenizer (Ultra-Turrax T25; IKA). Isolation of RNA, cDNA synthesis, and real-time RT-PCR were performed as described elsewhere [18]. For mouse mast cell protease (Mcpt)-1, Mcpt-5 and Mcpt-8, real-time PCR was performed with the iCycler (Bio-Rad) using SYBR Green (Applied Biosystems) for the detection of PCR products. Beta actin was used as house keeping gene. Primer sequences are available upon request. The relative expression was calculated by using the following formula:
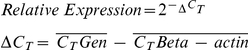



### Data analysis and statistics

All the data except the *in vivo* data are expressed as mean ± SEM. Data were analyzed for statistical significance using Prism 5 software (GraphPad Software, Inc.). Student's t tests (paired or unpaired as appropriate) were applied in a two-group analysis. Differences between the means of multiple groups were analyzed by the one-way analysis of variance, followed by a Tukey's multiple comparison test. Differences among both groups in the *in vitro* experiments employing combined hormonal treatment was analyzed by Mann-Whitney-U test. The *in vivo* data are expressed as dot plots showing median and differences were analyzed by Kruskal-Wallis test followed by Mann-Whitney-U test among two groups. In all cases, p<0.05 was considered significant and was the threshold to reject the null hypothesis.

## Results

### Mast cells strongly interact with trophoblast cells

**Figure 1:**
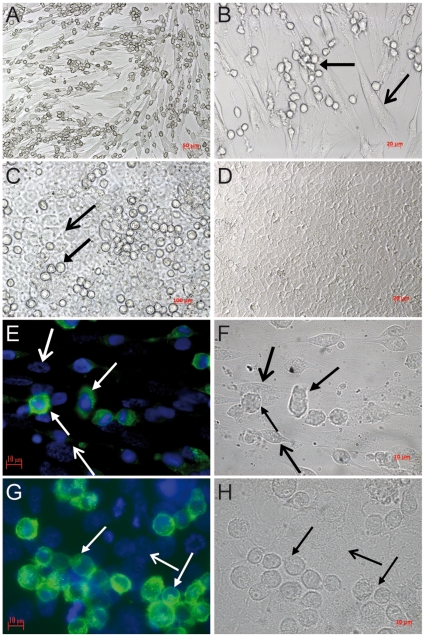
MCs and human trophoblast cells strongly interact with each other. Co-culture system between primary first trimester trophoblast cells or choriocarcinoma trophoblast cell line (JEG-3) with HMC-1 MC line (Fig. 1A–B and Fig. 1C respectively). Big adherent cells represent trophoblasts (indicated with an open arrow) whereas smaller, round cells attached to them are MCs (indicated with a closed arrow). As negative control we co-cultured a human keratinocyte cell line (HaCaT) with HMC-1 cells. Fig. 1D shows the complete absent of HMC-1 cells attached to the HaCaT cell after washing. Fig. 1E and Fig. 1G show HMC-1 CD117^+^ cells as stained by immunofluorescence co-cultured either with human trophoblasts or JEG-3 cells respectively. F and H pictures were done by light microscopy on the same area than Fig. E and G respectively DAPI was used as counterstaining. Fig. 1A, C, D were done with a 200× total magnification; Fig. 1B was done with a 400× total magnification and Fig. F, H were done with a 1000× total magnification under light microscopy using the Axiovision Rel 4–6 program (Zeiss AX 10 microscope). Fig. E, G were done with a 1000X total magnification by using the HXP-120 Light Source for Fluorescence Illumination and the Axiovision Rel 4–6 program (Zeiss AX 10 microscope).

The presence of MCs in the uterus and placenta as well as their interaction with the pre-implantation embryo and trophoblast cells were already described [3]. Moreover, it is known that trophoblast cells induce the release of histamine from uterine MCs by secreting histamine releasing factor [4]. In this work, we developed a co-culture system aimed to analyze in greater detail the interaction between both cell types. We co-cultured either human first trimester trophoblasts or JEG-3 cells both growing attached to the bottom of the culture flask together with HMC-1 which grow in suspension. As control we included a human keratinocyte cell line (HaCaT) which was co-cultured with HMC-1 cells under the same conditions. After 24 h of co-culture the supernatant was removed and the attached cells were washed twice with PBS. Surprisingly, even after washing, a high number of HMC-1 cells remained strongly attached to both human first trimester trophoblast and JEG-3 cells (Fig. 1A, B, C) while no HMC-1 cells remained adherent to HaCaT cells after washing (Fig. 1D). To further confirm our observations, after 24 h of co-culture, free-floating cells were washed and the attached cells were fixed. Immunofluorescence was performed in order to detect MCs attached to either trophoblasts or JEG-3 cells by using an antibody against CD117, which is a marker for MCs. We confirm that in fact HMC-1 cells strongly attach to both, human trophoblasts and JEG-3 cells as shown in Fig. 1E-F and G-H respectively.

### MCs actively migrate towards trophoblast and uterine cells

**Figure 2:**
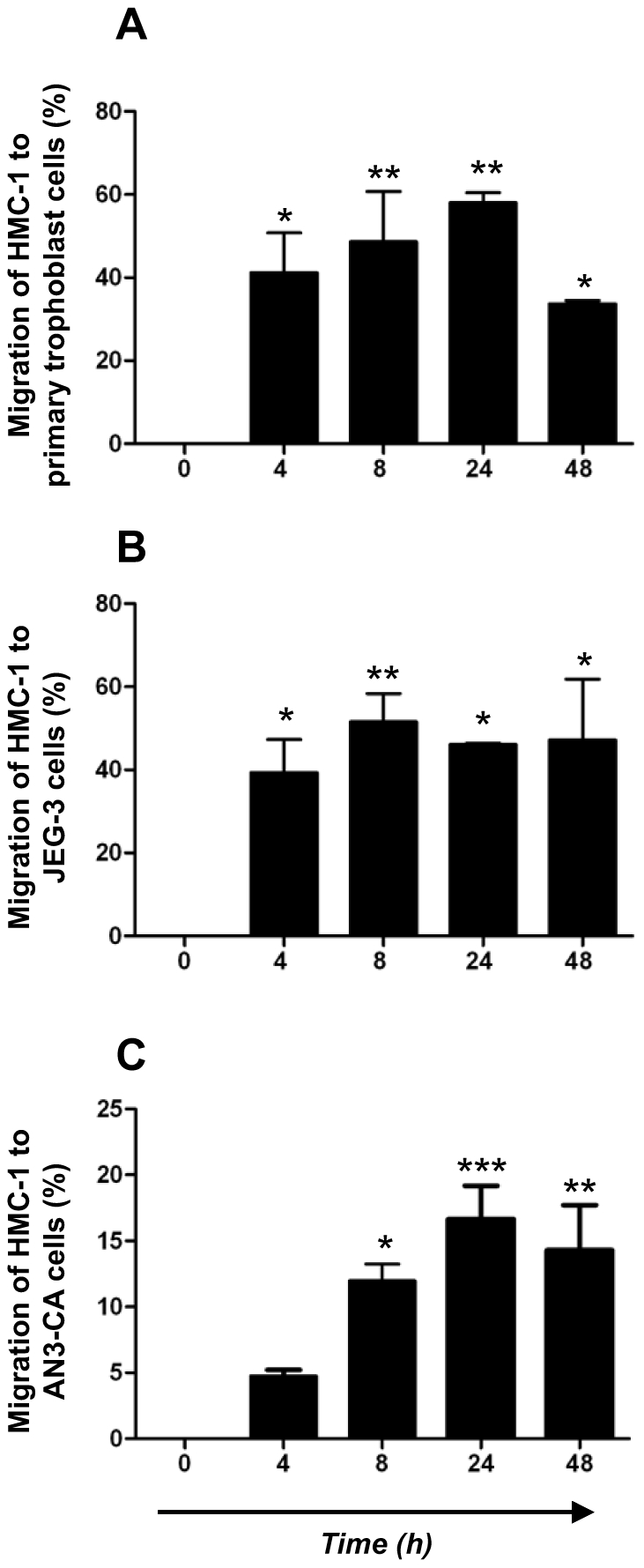
Human MCs actively migrate towards human trophoblast cells as well as to uterine cells treated with hormones. Using a two-chamber trans-well system, the migration of HMC-1 cells to primary first trimester trophoblasts (Fig. 2A) or JEG-3 cells (Fig. 2B) as well as to uterine cell line (AN3-CA) (Fig. 2C) upon hormonal treatment was analyzed at different time points (0, 4, 8, 24, and 48 h) by determining the relative number of HMC-1-CD117^+^ cells present in the lower part of the system referred to the total number of CD117^+^ cells in the upper and lower chambers. Spontaneous migration (ca. 5%) was subtracted for all time points. Data are representative of four experiments done in duplicates each. *:p<0.05 and **:p<0.01 as analyzed by ANOVA test followed by Tukey's test.

After observing MCs strongly attached to both, human primary first trimester trophoblast and JEG-3 cells, we next investigated whether soluble factors released from trophoblasts may attract human MCs. We therefore performed migration assays by using the well-documented transwell method between HMC-1 cells on the upper side and human primary trophoblast cells or JEG-3 cells in the bottom, both separated by a 8 µm thick transwell. As shown in Fig. 2, HMC-1 cells strongly migrated towards both, human first trimester trophoblast cells (Fig. 2A) and JEG-3 (Fig. 2B) cell line. After 4 h a migration of 40% can be observed, while the highest percentage of migration was observed after 24 h and toward primary trophoblast cells (Fig. 2A). This point out that trophoblasts actively attract MCs. This may occur under hormonal regulation as the placenta is a main source of estrogen and progesterone. To understand whether MCs are also attracted to uterine tissue after hormonal changes, e.g. during menstrual cycle, we additionally tested the capacity of the uterine cells to induce the migration of MCs under hormonal influence. We stimulated AN3-CA cells with E_2_ and P_4_ and analyzed the migration of HMC-1 cells by using migration assay. HMC-1 cells strongly migrated toward E_2_ + P_4_-treated human uterine cells (AN3-CA) as shown in Fig. 2C. The highest percentage of migration was observed after 24 h of culture (22%). Our data confirm that MCs can migrate to both, uterus and fetal-maternal interface. We next concentrated on the mechanisms of migration of MCs to the uterus and fetal-maternal interface upon hormonal influences.

### Estradiol and progesterone regulate in vitro the expression of chemokine receptors CCR4 and CCR5 in HMC-1 cell line as well as CCR3 and CCR5 in BMMCs

**Figure 3:**
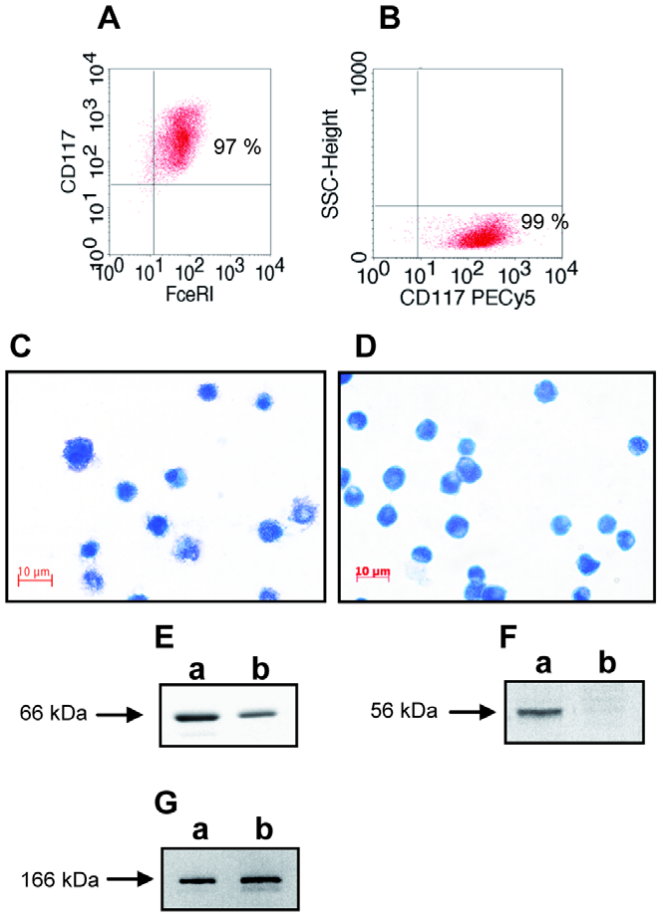
BMMCs and HMC-1 express high levels of CD117 as well as estradiol and progesterone receptors. Dot plots of cultured BMMCs (A) or HCM-1 cells (B) stained for CD117/FceRI as and CD117 as analyzed by flow cytometry. (C) BMMCs and HCM-1 cells (D) were stained with toluidine blue and they present typical features of MCs as analyzed by light microscopy using a total augmentation of 1000 X (Zeiss AX 10/Axiovision Rel 4.6). (E) and (F) represent western blots for estrogen receptor (ERα) and beta (ERβ) while (G) represent western blot for progesterone receptor (PR), respectively for HCM-1 (a) and BMMCs cells (b). β-actin served as house keeping gene.

**Figure 4:**
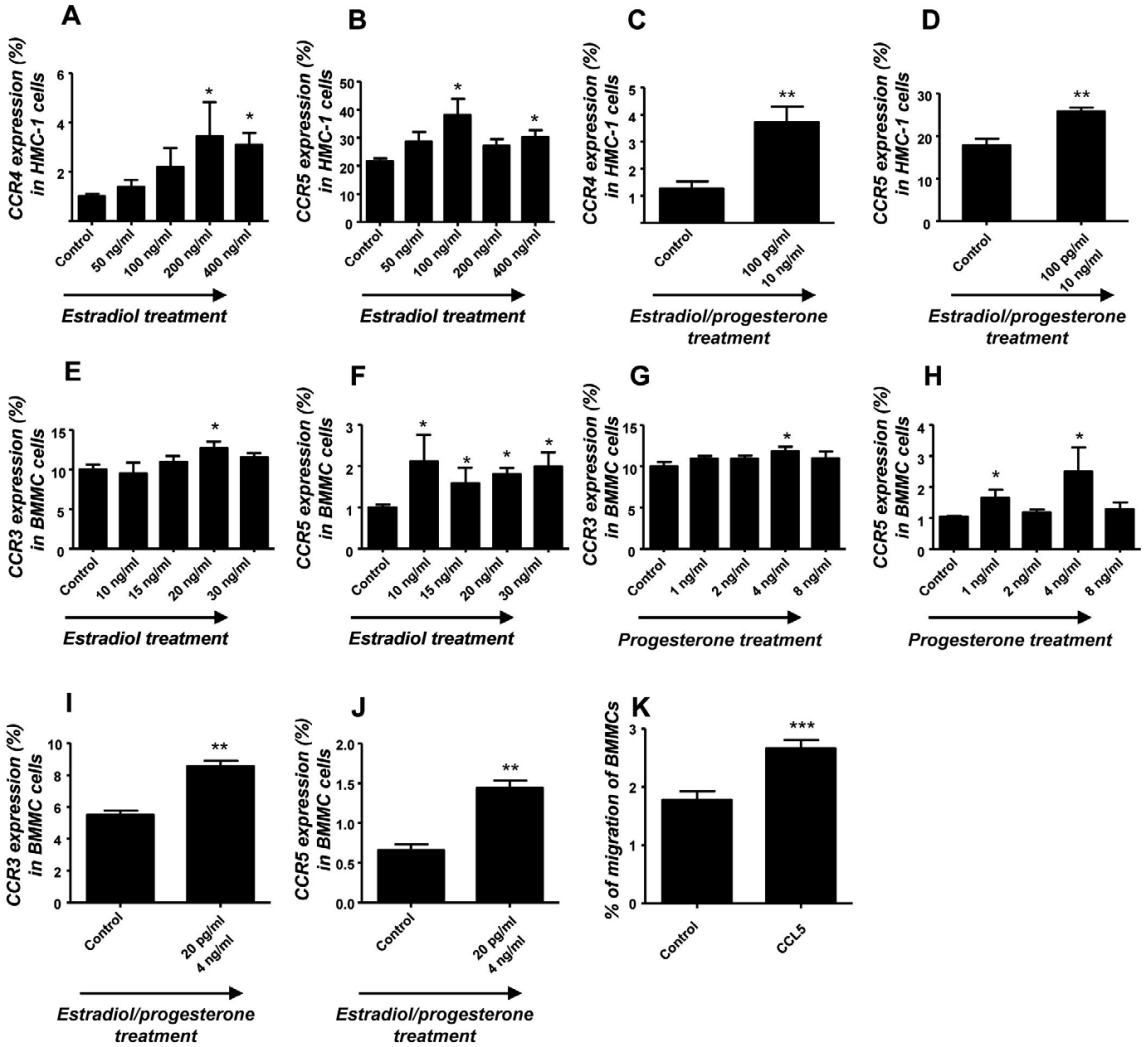
Estradiol and progesterone treatment in vitro results in the up-regulation of chemokine receptors in MCs. A–B: Chemokine receptor expression CCR4 (A) or CCR5 (B) on HMC-1 cells treated for 1 h with different physiological concentrations of estradiol (E_2_). E_2_ significantly up-regulated the expression of CCR4 and CCR5 in HMC-1 cells. C-D: Chemokine receptor expression CCR4 (C) or CCR5 (D) on HMC-1 cells treated for 1 h with a combination of E_2_P_4_. E_2_P_4_ treatment induced a significant up-regulation of both, CCR4 and CCR5 on HMC-1. E–H: Chemokine receptor expression CCR3 (E and G) or CCR5 (F and H) on BMMCs treated for 1 h with different physiological concentrations of E_2_ (E and F) or progesterone (P_4_; G and H). Both hormones induced an up-regulation of CCR3 (E, G) and CCR5 (F, H) in BMMCs after 1 h of treatment. I–J: Chemokine receptor expression CCR3 (I) or CCR5 (J) on BMMCs treated for 1 h with E_2_P_4_. Treatment of BMMCs with a combination of both hormones induced a significant increment on the expression of CCR3 and CCR5. The data are representative of four experiments performed in duplicates or triplicates. Chemokine receptor expression was analyzed by flow cytometry and data are expressed as percentage of cells expressing the chemokine receptors analyzed as mean ± S.E.M. *:p<0.05 as analyzed by ANOVA test followed by Tukey's test (A–B, E–H). The Mann-Whitney-U test for two particular groups was used (C–D, I–J). In all cases the expression of chemokines receptors for each dose vs. control was compared. K: BMMCs migration toward CCL5 (3 hours) is expressed as percentage of cells that migrated towards the CCL5 gradient from the total amount of cells. Spontaneous migration was subtracted. The data are representative of three experiments and are expressed as means ± S.E.M. *:p<0.05, **: p<0.01 and ***: p<0.001 as analyzed by the Mann-Whitney-U test.

It is known that MCs exist in the periphery as precursor cells and migrate to the tissues where they undergo their maturation upon different signals [25]. It has also been reported that the number of MCs oscillate in the uterus of mice throughout estrus cycle [26]. We confirmed that BMMC cultures which contain >97% pure mast cells (MCs) after 5 weeks (Fig. 3A–D) express both estradiol and progesterone receptor (Fig. 3E–G) and that they migrate to uterine cells upon hormones (Fig. 2C). Uterine cells both, in human and mouse release chemokines under hormonal influence throughout menstrual and estrus cycle [15]–[17]., We next analyzed the ability of physiological concentrations of E_2_ and P_4_, to modulate the expression of chemokine receptors in the immature human mast cells HMC-1 as well as in mouse bone marrow-derived MCs (BMMCs) as a possible mechanism for MC migration. As previously reported, HMC-1 and BMMCs express both, E_2_ and P_4_ receptors [27], [28], [24] (Fig. 3E–G). We then treated HMC-1 cells with 50, 100, 200 and 400 ng/ml of E_2_ and with 1, 5, 10 and 50 pg/ml of P_4_ which represent the physiological concentrations of these hormones during the female menstrual cycle in humans. Additionally we treated HMC-1 cells with a combination of both hormones at their most effective concentration (100 pµ/ml-10 ng/ml). We analyzed the expression of CCR4 and CCR5, which represent the most important receptors for chemokines known to be released from the uterus under hormonal influence in humans [15]. As shown in Fig. 4A and 4B, E_2_ concentrations of 200 and 400 pg/ml or 100 and 400 pg/ml, corresponding to middle cycle and early luteal phase significantly up-regulated the expression of CCR4 and CCR5 respectively on HMC-1 cells after 1 h of treatment. Physiological concentrations of P_4_ did not affect the expression of these chemokine receptors on HMC-1 cells (data not shown). Furthermore, when HMC-1 cells were treated with a combination of both hormones, a significant up regulation of CCR4 and CCR5 was observed (Fig. 4C, D). In mice, the most important chemokine receptors for the chemokines released from the uterus are CCR3 and CCR5 [16]–[17]. BMMCs were stimulated with physiological concentrations of E_2_ (10, 15, 20 and 30 pg/ml), P_4_ (1, 2, 4, 8 ng/ml) or a combination of both (20 pg/ml +4 ng/ml), which mimic the concentrations observed during estrus cycle [29] and after 1 h of treatment the expression of CCR3 and CCR5 in BMMCs was analyzed by flow cytometry. As shown in Fig. 4E–H both, E_2_ and P_4_ significantly up-regulated the expression of CCR5 and CCR3. Moreover, a combination of both hormones significantly induced the expression of CCR5 and CCR3 in BMMCs after 1 h of treatment (Fig. 4I–J). Accordingly, CCL5 (RANTES) is able to attract murine MCs (Fig. 4K), which express both CCR3 and CCR5. Thus, CCR4 and CCR5 in humans and CCR3 and CCR5 in mice are capable to mediate MC migration upon hormone signals.

### Estradiol and progesterone induce the maturation of MCs as well as their degranulation

**Figure 5:**
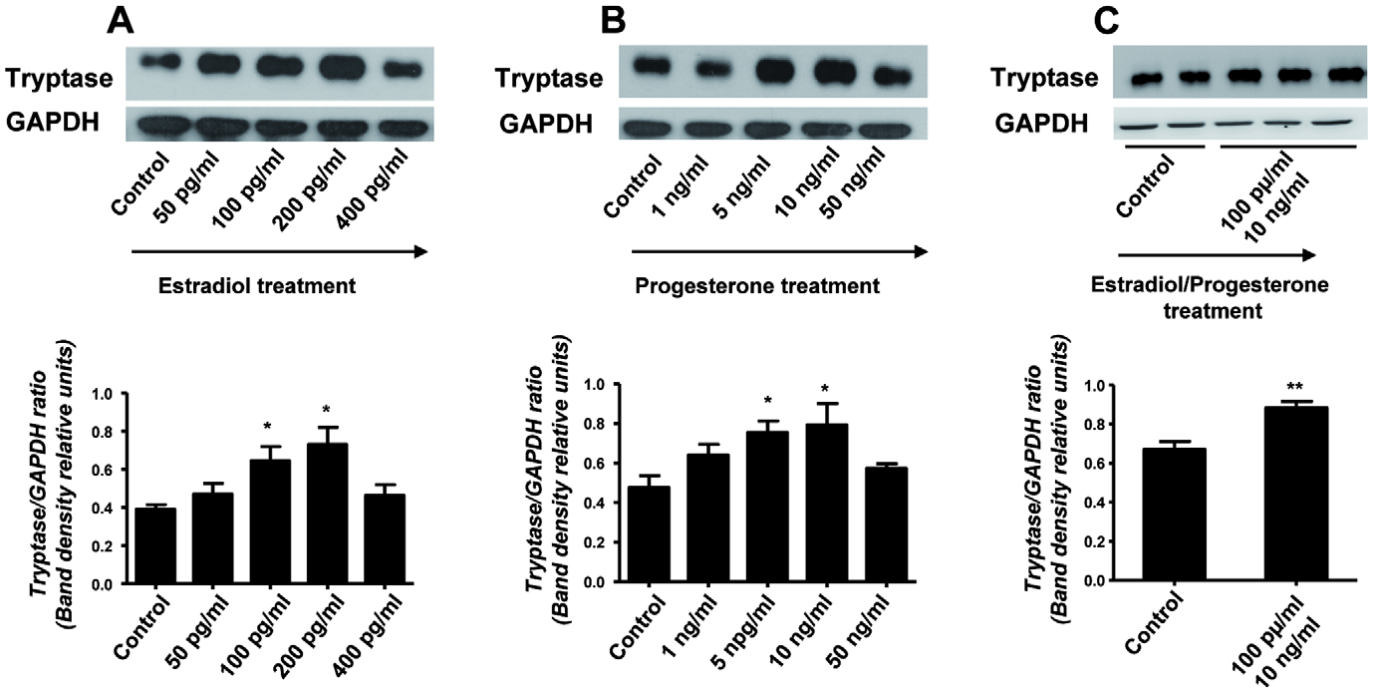
Estradiol and progesterone treatment in vitro as well as their combined application up-regulate the expression of human tryptase in HMC-1 cells. Human mast cell tryptase expression on HMC-1 cells treated for 1 h either with estradiol (E_2_; A), progesterone (P_4_; B) or (E_2_ + P_4_; C) was analyzed by Western Blot using GAPDH as house keeping gene. Physiological concentrations of both estradiol (A) and progesterone (B) corresponding to their concentrations towards middle menstrual cycle or just after ovulation takes place respectively as well as the combination of both hormones at their most effective concentration, significantly up-regulated the expression of human tryptase on HMC-1 cells (C). Data are expressed as Tryptase/GAPDH ratio (mean ± S.E.M.) *:p<0.05 as analyzed by ANOVA test followed by Tukey's test (A–B) or The Kruskall-Wallis test followed by Mann-Whitney-U test (C). Each experiment was done in triplicate; the blots are representative from three independent experiments. Tryptase expression in HMC-1 cells after hormonal treatment was compared to the controls in each case.

**Figure 6:**
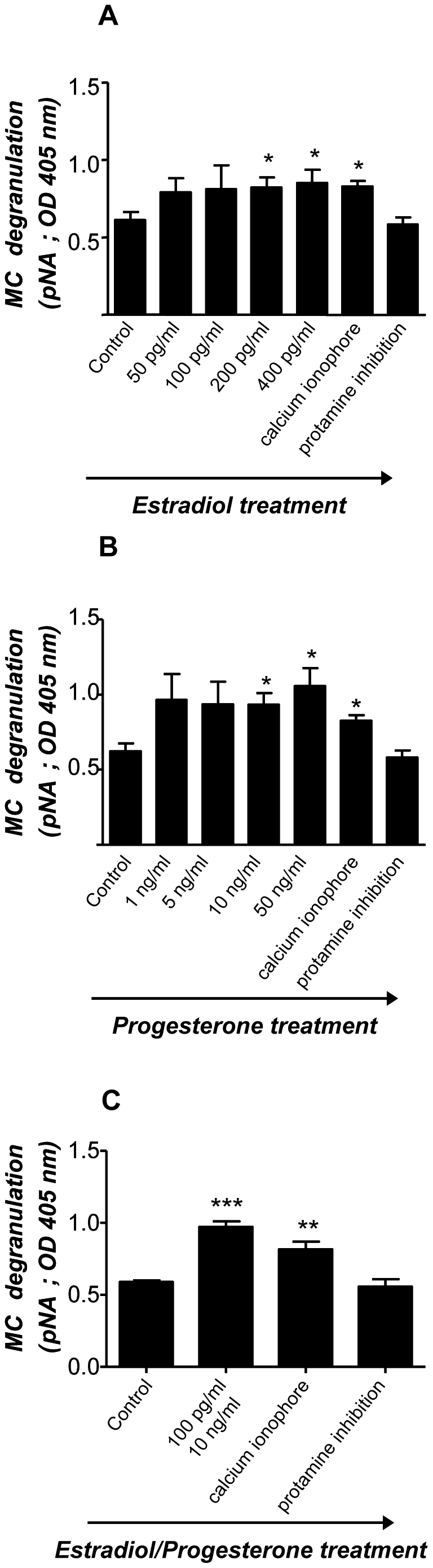
Estradiol and progesterone as well as a estradiol-progesterone combined treatment induce in vitro HMC-1 degranulation. Physiological concentrations of both, estradiol (E_2_; A) and progesterone (P_4_; B) significantly induced HMC-1 degranulation after 1 h of treatment as analyzed by measuring chromophore p-nitroaniline (pNA) produced after cleavage from the labeled substrate tosyl-gly-pro-lys-pNA by tryptase present in the supernatant of HCM-1 cells employing a MC-degranulation kit. (C) shows the degranulation observed after *in vitro* application of both, estradiol and progesterone. Positive controls included the treatment of HMC-1 cells with calcium ionophore to induce degranulation while negative controls were cells treated with calcium ionophore plus protamine inhibitor. Data are expressed as mean ± S.E.M of the optical density at 405 nm as measured in the supernatant of treated cells. *:p<0.05, **:p<0.01 and ***:p<0.001 as analyzed by ANOVA test followed by Tukey's test. Each experiment was done in triplicate.

After confirming that hormones are involved in the expression of chemokine receptors on MCs, which may provide a mechanism as to why they migrate to the uterus, we further investigated whether E_2_ and P_4_ have also an influence on MC maturation and degranulation. As a maturation marker we analyzed the expression of human tryptase, which is the most abundant secretory granule-derived serine proteinase contained in MCs [30]. As shown in Fig. 5, both E_2_ (A) and P_4_ (B) significantly up-regulated the expression of human tryptase in HMC-1 cells. Treatment of HMC-1 cells with a combination of both hormones E_2_ + P_4_ clearly showed a statistically significant augmentation on the expression of human tryptase (Fig. 5C). This clearly shows the positive effects of female hormones on MC maturation. Because MC degranulation implies the release of pivotal factors involved in embryo implantation [31]–[32], we subsequently analyzed the influence of physiological concentrations of E_2_ and P_4_ on MC degranulation. Concentrations of E_2_ and P_4_ similar to those described for the analysis of chemokine receptors were employed to stimulate HMC-1 cells and their degranulation was measured. Both, E_2_ and P_4_ significantly induced HMC-1 degranulation in a dose-dependent manner. Concentrations of E_2_ similar to those observed in humans during middle cycle, thus after ovulation, (200 and 400 pg/ml) significantly induced HMC-1 degranulation (Fig. 6A). P_4_ concentrations comparable to values observed systemically during embryo implantation and early gestation (10 and 50 ng/ml) significantly induced degranulation of HMC-1 cells as well (Fig. 6B). Additionally, treatment of HMC-1 cell with E_2_ + P_4_ induced a significant degranulation of HMC-1 cells compared to the levels observed when each hormone was administrated separately. Our results reveal that female hormones can induce both, MC maturation and degranulation *in vitro*. Because the factors released by MCs belong to the family of mediators involved in embryo implantation [31]–[32], we propose a scenario in which hormones attract MCs to the uterus during menstrual cycle and once there promote their maturation and degranulation, which would prepare the uterus for a possible embryo implantation.

### E_2_ and P_4_ treatment induce the in vivo migration of MCs from the periphery to the uterus of ovariectomized mice as well as their maturation and degranulation

**Figure 7:**
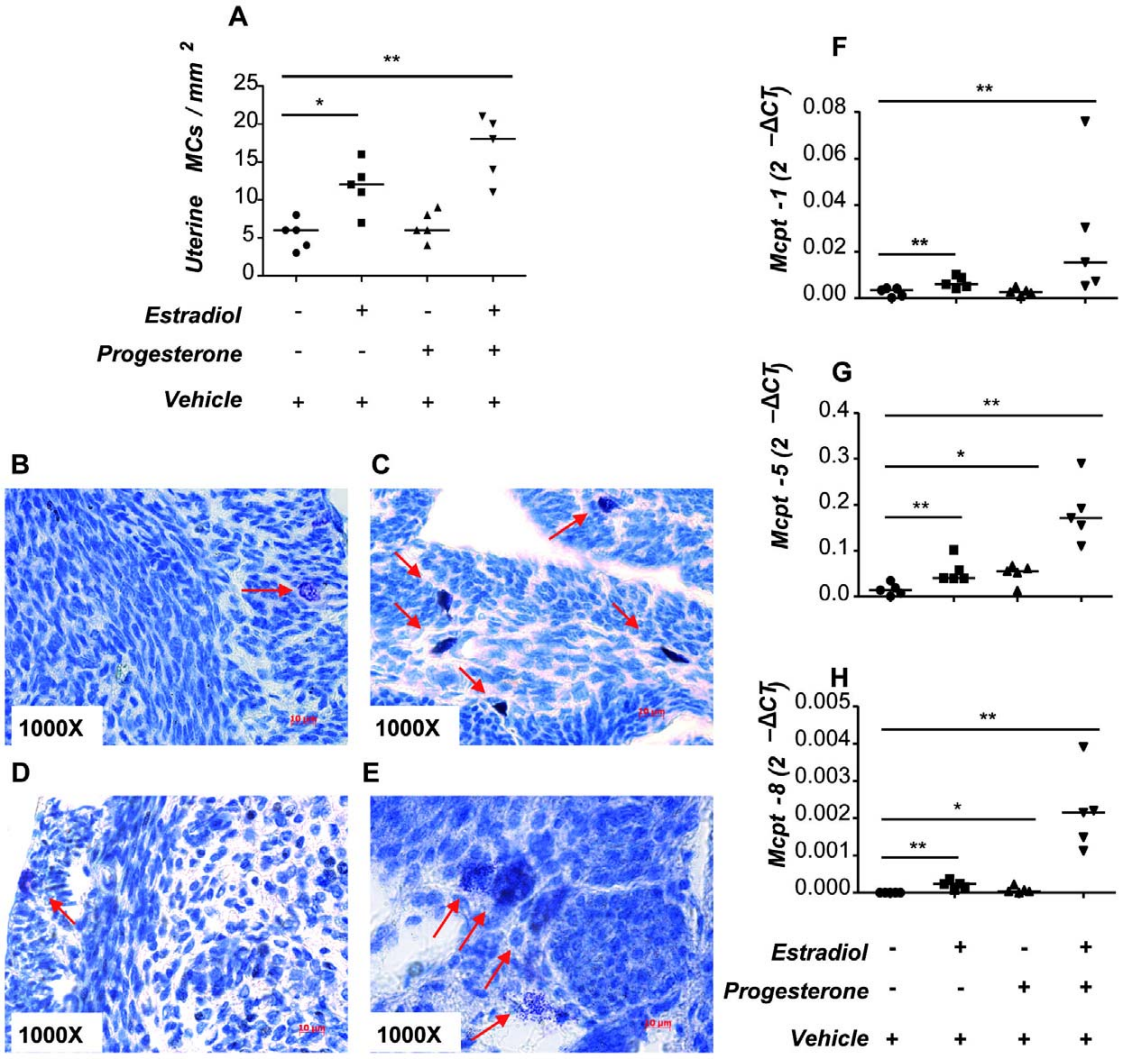
Female hormones induce the migration of MCs to the uterus, their maturation and degranulation Virgin ovariectomized C57BL/6J mice were treated with estradiol (E_2_), progesterone (P_4_) or estradiol + progesterone (E_2_ + P_4_). The number of MCs was quantified in 10 fields of uterine tissue from all animals (n = 5/group). MC visualization was performed by tolouidine blue staining and quantification was done using a total magnification of 1000 X under light microscopy using the Axiovision Rel 4–6 program (Zeiss AX 10 microscope). E_2_ alone or in combination with P_4_ induced a significantly augmentation in the number of uterine MCs as compared to the vehicle-treated animals (A). Representative pictures from uterine MCs from control (B), E_2_-treated (C), P_4_-treated (D) and E_2_ + P_4_- treated animals (E) are shown. Arrows indicate uterine MCs, which are easily distinguishable because of their granula. F-H: show the expression of MC-related genes Mcpt-1 (F), Mcpt-5 (G) and Mcpt-8 (H) in uterus of treated and control animals as analyzed by real time RT-PCR. The results are expressed as single dots showing medians. *:p<0.05 and **:p<0.01 as analyzed by Kruskall-Wallis test followed by Mann-Whitney-U test for two particular groups.

After observing that female hormones participate in the maturation and degranulation of MCs and that these cells actively migrate to trophoblasts, which produce E_2_ and P_4_, we postulate that these hormones may also promote the migration of MCs or their precursors to the uterus *in vivo*. For this, we took advantage of an *in vivo* model consisting of ovariectomized (OVX) mice, in which female hormones, E_2_ and P_4_, are almost absent [33]. We quantified the number of MCs present in uterine tissues and analyzed whether the application of hormones would mobilize MCs or their precursors from the periphery to the uterus as well as induce their maturation and subsequent degranulation. OVX mice were therefore treated with E_2_, E_2_ + P_4_ or P_4_ for 3 consecutive days following published protocols [33], [34]. After that, animals were sacrificed and the numbers of MCs present in the uterus, the percentage of degranulation and the expression of MC related genes were investigated. MC degranulation was studied microscopically by quantifying secreted granules. MC presence was confirmed in uterus of OVX vehicle-treated animals (Fig. 7B) and the percentage of MC degranulation was calculated (19.3±5.17%). E_2_ application induced a significant augmentation in the number of uterine MCs (Fig. 7A) while did do not significantly affect the percentage of MC degranulation (15.21±4.34% p = 0.16) when compared to the vehicle-treated animals. P_4_ application did not induce any significant changes either in the number (Fig. 7A) or in the percentage of MC degranulation (22.14±3.71% p = 0.21) as compared to the controls. Treatment of OVX animals with a combination of both, E_2_ and P_4_ provoked profound changes in MC number (Fig. 7A, E, Fig. S1). Not only significantly more MCs were present in the uterus as compared to vehicle-treated or E_2_-treated animals but also a positive effect on degranulation was observed (50.33±9.87% ** p = 0.0097) after combined hormone treatment. We further observed that the combined application of E_2_ and P_4_ significantly induced the expression of Mcpt-1, Mcpt-5 and Mcpt-8 mRNA as compared to the vehicle-treated group (Fig. 7G, H). This confirms the positive effect of these hormones on MC maturation and degranulation. Our *in vivo* data clearly support the hypothesis postulated after the *in vitro* observations and confirm that hormonal changes during both, menstrual or estrus cycle, attract MCs to the uterus. Female hormones also stimulate MC maturation and degranulation, which may prepare the uterus for a possible embryo nidation and pregnancy.

## Discussion

There are strong evidences linking female sex hormones with mast cells (MCs). For instance, asthma and other diseases involving MCs have higher incidence in women than in men during early to middle adulthood [35]–[37]. Several clinical and epidemiological studies have pointed out the role of female hormones on these differences. Moreover, it has been shown that postmenopausal women receiving hormone replacement therapy have higher risk of new onset of asthma [27]. Additional data supporting this finding is the fact that 30–40% of women who have asthma, experience worsening of their symptoms during the perimenstrual phase (perimenstrual asthma), when estrogen and progesterone concentrations are changing rapidly [38]. The presence of MCs in the uterus has been already described in many species including human [39] and MC number has been found to fluctuate during estrous cycle in rats [40], mice [25], guinea pigs [41] and cows [42], suggesting an influence of female hormones on MC recruitment to the uterus. However, whether this is the case and the mechanisms behind MC migration from the periphery to the uterus remain unclear. Herein, we provide evidence that MCs migrate from the periphery to the uterus under hormonal influence and promote their maturation and degranulation in uterine tissue. We further show that the chemokine receptors CCR4 and CCR5 in human and the chemokine receptors CCR3 and CCR5 in mouse may be involved in MC recruitment to the uterus after hormonal changes.

We began analyzing whether MCs and trophoblast cells, which are known to produce both, female hormones and chemokines, interact with each other. We were able to confirm that MCs strongly adhere to trophoblast cells when co-cultured and that MCs can actively migrate to primary trophoblasts and to JEG-3 trophoblast cells. Accordingly, they migrate to hormone-treated uterine cells. *In vivo*, estradiol and progesterone treatment in OVX mice which are not able to produce these hormones clearly induced the migration of MCs to the uterus and the posterior maturation and degranulation of these cells. This irrefutably confirms MC migration to the uterus/fetal-maternal interface upon hormonal changes.

The human uterus produces and releases several chemokines throughout the menstrual cycle and during early pregnancy, which are under hormonal control [15]. A similar scenario occurs in the mouse [16]–[17]. It is known that chemokines are critical for leukocyte recruitment, activation and homing into different tissues. MCs from peripheral blood migrate into tissues as committed progenitors and develop into mature cells *in situ* [24]. They migrate within peripheral tissues mainly in response to locally produced chemokines [43]. We therefore hypothesized that female hormones may be in charge of recruiting MCs into the uterus because of their ability to modulate the production and release of chemokines from the uterus. After confirming that CCL5, one potent chemokine with various receptors, can in fact attract MCs, we next tested whether hormones are also able to modulate the expression of its chemokine receptors on MCs, which would allow the migration of MCs to the uterus upon chemokine gradient. We studied the implication of estradiol and progesterone treatment on the expression of CCR5, CCR4 and CCR3 which represent the most important chemokine receptors for the chemokines released from the uterus (CCL7, CCL22, CCL5, CCL4, CCL11) [15]–[17]. We did so by utilizing the HMC-1 human mast cell line as well as mouse bone marrow-derived mast cells (BMMCs). Estradiol, but not progesterone did stimulate the expression of CCR5 and CCR4 protein on HMC-1 while both, estradiol and progesterone were able to significantly up-regulate the expression of CCR5 and CCR3 on BMMCs. The fact that only estradiol but not progesterone induced a modulation of chemokine receptors in the HMC-1 is in accordance with the hormonal pattern during menstrual cycle in human. Estradiol is the dominant hormone during the first half of the female cycle (follicular phase), while progesterone is almost absent. Once ovulation takes place, progesterone becomes the main hormone (secretory phase). Hence, progesterone may be more important for maturation and degranulation of human MCs rather on their recruitment, which is on line with our results concerning the effects of progesterone on maturation and degranulation. Unlike HMC-1, in BMMCs, both estradiol and progesterone had stimulatory effects on CCR5 and CCR3 expression. These results are also in accordance with the hormonal pattern during the estrus cycle in mice [44]. Both hormones are present throughout the whole estrus cycle; hence, they exert a combined effect on the expression of the chemokine receptors in mouse MCs.

After confirming that estradiol and progesterone and also a combination of both regulate the migration of MCs to the uterus we wondered which would be their function on this tissue. Embryo implantation is a pivotal step during pregnancy and the failure of the embryo to implant into the uterine endometrium at early pregnancy stages is a major cause of infertility [45]. Implantation involves embryo apposition and adhesion to the endometrial epithelium followed by penetration through the epithelium and invasion of the embryonic trophoblast through the endometrial stroma, which require extra cellular matrix degradation [46]. MCs were proposed to be important players on embryo implantation [30]–[32] as their degranulation leads to the release of pivotal factors, e.g. histamine, tryptases, MMPs, VEGF, [2], [30], [45], [47]. These factors are crucial for extracellular matrix degradation and neo-vascularization, both processes necessary for the correct attachment of the embryo to the uterus and subsequent embryo development. Matrix metalloproteinases (MMPs) were proposed as key regulators in the capacity of the trophoblast cells to invade the uterine wall. MMPs are produced by cytotrophoblastic cells (CTB) and are instrumental in their invasive behavior. Zhang and co-authors showed that MCs present in the human uterus are able to induce the expression of MMPs both, in endometrial stromal and decidual cells [48]. Furthermore, MC tryptase has remarkably restricted substrate specificity for proteins, their substrates include proMMP-3 [49], urokinase type plasminogen activator [50], collagen VI [51], and a1 macroglobulin [52], while chymase is able to activate proMMP-1 [53]. Another pivotal process during embryo implantation and posterior development is the formation of new blood vessels which support embryo growth. Angiogenesis occurs regularly in association with cyclical changes in adult female endometrium and also during implantation and subsequent placentation [54]. In rat, it has been shown that the active angiogenic process that takes place in the uterine cervix during gestation is regulated by secretion of contents stored in MCs granules [24]. Moreover, the proangiogenic factor VEGF released from MCs after MC degranulation was implied as a key regulator on this process [47].

Here, we demonstrated that estradiol and progesterone attract MCs into the uterus and fetal-maternal interface and also have a positive effect on MC maturation and degranulation. Hormones attract MCs by modulating the expression of chemokine receptors on their surface. Our data bring to light novel mechanisms as to how MCs may migrate from the periphery to the uterus and how hormones modulate MC maturation and degranulation which would prepare the uterus for a possible embryo implantation.

## Supporting Information

Figure S1 MCs in the uterus of E2 + P4 treated animals (A–B) and control animals (C) were immunolocalized by CD117 immunofluorescence.
(0.27 MB TIF)
