# Supplementary figures and images for: Estradiol and Progesterone Regulate the Migration of Mast Cells from the Periphery to the Uterus and Induce Their Maturation and Degranulation
Source: PLoS One. 2010 Dec 22;5(12):e14409. doi: 10.1371/journal.pone.0014409 (PMC3008683; doi:10.1371/journal.pone.0014409)

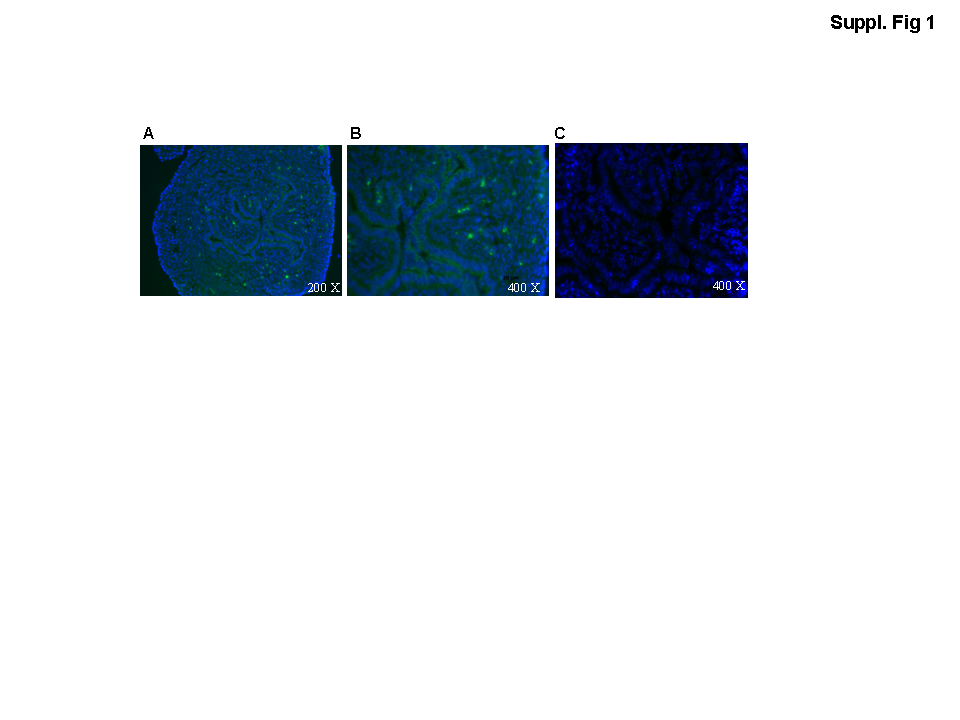

Supplement: Figure S1 — MCs in the uterus of E2 + P4 treated animals (A–B) and control animals (C) were immunolocalized by CD117 immunofluorescence. (0.27 MB TIF) [file pone.0014409.s001.tif]
